# Conditioning Program Prescribed from the External Training Load Corresponding to the Lactate Threshold Improved Cardiac Function in Healthy Dogs

**DOI:** 10.3390/ani12010073

**Published:** 2021-12-30

**Authors:** Alejandro Zamora Restan, Aparecido Antonio Camacho, Evandro Zacché, Raphaela Arantes Marques Canola, Samara Beretta Gomes Silva, Jaislane Bastos Braz, Jorge Cardoso da Silva-Filho, Juliana Aparecida Cerqueira, Bruna Agy Loureiro, Michelli Inacio Gonçalves Funnicelli, Daniel Guariz Pinheiro, Guilherme Camargo Ferraz

**Affiliations:** 1 Department of Animal Morphology and Physiology, Laboratory of Pharmacology and Equine Exercise Physiology (LAFEQ), School of Agricultural and Veterinarian Sciences (FCAV), São Paulo State University (UNESP), Jaboticabal 14884-900, São Paulo, Brazil; alejomvz1208@gmail.com (A.Z.R.); saberetta11@hotmail.com (S.B.G.S.); julianacerqueira.vet@gmail.com (J.A.C.); 2 Department of Clinics and Surgery, School of Agricultural and Veterinarian Sciences (FCAV), São Paulo State University (UNESP), Jaboticabal 14884-900, São Paulo, Brazil; aa.camacho@unesp.br (A.A.C.); ezacche@yahoo.com.br (E.Z.); raphaelamarques@hotmail.com (R.A.M.C.); jaislanemedvet@gmail.com (J.B.B.); filhojcs@hotmail.com (J.C.d.S.-F.); 3 Department of Animal Science, Federal University of Paraiba, Paraiba 58051-900, Brazil; brunaagy@yahoo.com.br; 4 Department of Agricultural, Livestock and Environmental Biotechnology, School of Agricultural and Veterinarian Sciences (FCAV), São Paulo State University (UNESP), Jaboticabal 14884-900, São Paulo, Brazil; michelli.funnicelli@unesp.br (M.I.G.F.); daniel.pinheiro@unesp.br (D.G.P.)

**Keywords:** exercise, anaerobic threshold, left ventricle, strain, echocardiography, principal component analysis, heat map

## Abstract

**Simple Summary:**

Regular exercise is a stressful stimulus that elicits physiological responses in systolic and diastolic functions in human athletes, the so-called “athlete’s heart”. The present study reports findings obtained from echocardiography to measure the ventricular dimensions at rest in beagle dogs undergoing an endurance training program carried out on a treadmill with the intensity set at 70–80% of the velocity corresponding to the lactate threshold. Echocardiography was performed with routine measurements of the left ventricular systolic and diastolic function by the two-dimensional and Doppler techniques. After the training, the principal component analysis of echocardiographic variables was conducted to evaluate dimensional changes in left ventricular function. Principal components analysis was able to capture the qualitative echocardiographic changes produced by the endurance training. Eight weeks of the lactate-guided endurance training program could lead to concomitant left ventricular dilation without hypertrophy of the ventricular walls, emphasizing the left ventricular systolic and diastolic functions. These results suggest that submaximal aerobic training may induce physiological cardiac remodeling, improve the left ventricular functions, promote health, and minimize any injuries produced during heart disease, although its effectiveness for the latter effect must be confirmed in future studies.

**Abstract:**

This research focuses on the adjustments in systolic and diastolic functions that are not fully understood in dogs submitted to athletic training. Beagle dogs carried out an endurance training program (ETP) prescribed from the external training load, corresponding to 70–80% of the lactate threshold (VLT) velocity. Eighteen dogs were randomly assigned to two groups: control (C, *n* = 8), active dogs that did not perform any forced exercise, and trained (T, *n* = 10), submitted to the ETP during eight weeks. All dogs were evaluated before and after the ETP period using two-dimensional echocardiography, M-mode, Doppler, and two-dimensional speckle tracking. A principal component analysis (PCA) of the echocardiographic variables was performed. The ETP improved the left ventricular internal dimension at the end of diastole (LVDd), the left ventricular internal dimension at the end of diastole to aorta ratio (LVDd: Ao), and the strain rate indices. PCA was able to capture the dimensionality and qualitative echocardiography changes produced by the ETP. These findings indicated that the training prescribed based on the lactate threshold improved the diastolic and systolic functions. This response may be applied to improve myocardial function, promote health, and mitigate any injuries produced during heart failure.

## 1. Introduction

Prolonged physical conditioning is often associated with inducing morphological and functional cardiac changes in human athletes, the so-called “athlete’s heart”. Cardiac remodeling in human athletes is well recognized. However, in veterinary medicine the approach has received less consideration [1]. Assessed by echocardiography, ventriculography, or nuclear magnetic resonance, this morphofunctional alteration may indicate an increase in the parasympathetic tone associated with an increase in the left and right ventricles. These physiological changes are also described in dogs used in various activities such as rescue, agility, or mushing [2,3,4]. Like the previous research in human athletes, evidence of large hearts in athletic horses antedate many years. Horses have been an important animal model for exercise physiology studies. Analyzing the association between heart size and aerobic performance in horses, the literature has shown intense positive relationships between several measures of cardiac function and $\dot{V}$O_2_max, a key decisive of aerobic capacity [1,5].

The cardiac remodeling response described in athletes such as cyclists [6], marathoners, and weightlifters [2,7] has been recently reported in dogs that practice bikejöring [2]. However, it must be distinguished from deleterious changes. This remodeling is characterized by an increase in the left ventricle internal dimension (LVID) in diastole, left ventricle (LV) hypertrophy, and left atrium increase (LA). LV adjustments are induced by volume overload associated with high cardiac output during acute and intense exercise. The increase in LA volume and dilation is caused by the compensatory response of the LA for maintaining the sound volume in the LV [8].

In recent years, considerable progress has been made in echocardiography techniques for detecting cardiac changes induced by regular exercise [2,9,10]. Noninvasive auxiliary diagnostic methods can determine these changes. In the literature, several studies revealed that the systolic function of rowers, marathoners, fighters, as well as active and healthy individuals improved with conditioning after being evaluated using myocardial deformation techniques such as speckle tracking [10,11,12]. Recently, echocardiography has been performed to assess the effects of overtraining syndrome in high-level male human athletes [13]. 

As a complex system, the organism acts as an inseparable and integrated whole that cannot be constrained only to the univariate quantitative statistical analysis of its physiological functions separately [14,15]. The cardiovascular system and the cooperative intrinsic function of its multiple subsystems are interdependent and interact in a dynamic and nonlinear way. Thus, the subsystems need to be approached as a nonlinear statistical design. These multivariate methodologies, focusing on the integrated aspect of cardiovascular variables in dogs, should consider the aerobic training effect on cardiovascular function. To this end, researchers have evaluated cardiorespiratory coordination in humans undergoing different training modalities by analyzing all the major components using a principal component analysis (PCA) approach. PCA diminishes the data dimensionality of associated systems, withdrawing the narrowest range of components that account for most of the variation in the seminal multivariate data and summarizing it with a minor deficit of information. This analysis may be used to monitor the integration of multiple variables in a physiological system and a wide range of biological research fields [15].

Prolonged physical exercise, if well prescribed, establishes forms of adjustment that enhance performance and may be called a “eustress”. “Eustress” suggests that an “acceptable or desirable stress degree” may have a positive influence on welfare and fitness [16]. The training load in the background of athletic training has been characterized as the input guideline manipulated to elicit the desirable physical conditioning response. Studies addressing the sport training protocols of human athletes often apply the external or internal load concept when elaborating and prescribing the training programs by considering the exercise’s volume, duration, and intensity. External load is defined as the variables that induce internal physiological responses such as duration, the distance covered, the average and maximum speeds, and the treadmill slope. Internal load indicates an effective physiological response promoted by the athlete to adjust to the stimuli induced by the external load. Both heart rate and plasma lactate concentration can be used as indicators to measure the internal load during running [17].

In studies with human athletes, exercise physiologists employ fitness protocols that were prescribed based on maximum oxygen consumption ($\dot{V}$O_2_max) and lactate threshold (LT) for specifying the training program load aimed at improving systolic and diastolic function in healthy or sick individuals [18,19,20,21]. In veterinary medicine, relatively few studies [22,23,24] have used the individualized prescription of internal or external load for elaborating training protocols while evaluating their effects on the echocardiographic variables. Moreover, prolonged submaximal exercise to prevent or treat cardiovascular disease is still relatively limited, notably in dogs. The benefits of prolonged training as a therapeutic measure to treat ventricular dysfunction diseases are practically not described in the specific literature on dogs [25]. Therefore, studies are needed to verify the safety and effects of the conditioning protocols performed with a refined control of the training load on healthy dogs. The scientific findings of these studies can be extrapolated to dogs with cardiovascular diseases.

Our research group recently determined the LT in dogs subjected to the incremental exercise testing (IET) [23,24,26] to obtain the velocity corresponding to the LT (VLT). This variable was the reference used for establishing the external training load since it is considered more reliable compared to both the percentage of maximum oxygen consumption (%O_2_MAX) and the maximum heart rate (%HRMAX) [16]. The training protocol improved aerobic capacity and increased heart rate variability after eight weeks of training [23,24].

To the best of the authors’ knowledge, no studies determining the cardiac function response of dogs submitted to the aerobic training prescribed from the VLT are available in the literature. Herein, we expand these findings by evaluating whether the endurance-type training, prescribed based on VLT, can improve healthy dogs’ diastolic and systolic functions. We also conducted a principal component analysis (PCA), a robust analysis tool for exploratory assessment and which consists of gaining observation in the degree of co-relatedness, or its absence, among the echocardiographic variables under examination.

## 2. Materials and Methods

### 2.1. Dogs

This was a prospective case–control study. The experiment was conducted in the Laboratory of Pharmacology and Physiology of Equine Exercise (LAFEQ) and the Cardiology Laboratory of “Governor Laudo Natel” Veterinary Hospital of São Paulo State University (UNESP), School of Agricultural and Veterinarian Sciences, Jaboticabal (FCAV), Brazil. The study followed the Ethical Principles in Animal Experimentation adopted by the Brazilian College of Animal Experimentation and approved by the Ethics Committee on Animal Use (CEUA), protocol 008272/17.

Eighteen healthy beagles (10 males and 8 females) aged 12 to 24 months were used in the study. The dogs belong to the kennel of the Laboratory of Nutrition and Nutritional Diseases of the School of Agricultural and Veterinarian Sciences (FCAV), Sao Paulo State University (UNESP), Jaboticabal, Brazil. All dogs lived in 1.5 × 4.0 m kennels with a solarium and 4 h daily access to the 1000 m^2^ outdoor area for spontaneous and accessible physical activity, recreation, and socialization. It is highlighted that the dogs were not submitted to any forced training on the treadmill before the experiment. The dogs were considered healthy based on their clinical history, physical examination, blood test, as well as a two-dimensional echocardiogram, M-mode, and Doppler. Exclusion criteria included any changes in blood tests and possible clinical abnormalities that could affect exercise performance, cardiovascular, neuromuscular, and orthopedic conditions.

The dogs were fed enough commercial food to satisfy their individual energy needs equivalent to the active dogs, according to the NRC [27]. Water was available ad libitum. During the trial, the dogs were fasted for a 12 h interval before training sessions or exercise mats but were continuously fed 20 min after the exercise sessions. On training days, food was given 20 min after the exercise period. During the experimental period, the dogs were weighed every fifteen days so that, when deemed necessary, the food amount was adjusted to maintain the body weight.

### 2.2. Experimental Groups

Enrolled dogs were randomly assigned to two groups: control (C, *n* = 8; 4 males, 4 females) consisting of active dogs that were not submitted to any training exercise, and the trained group consisting of dogs (T, *n* = 10; 6 males, 4 females) that were submitted to submaximal training on the treadmill three times a week for eight weeks. The control group was used to verify possible interference from environmental stimuli, such as behavioral and emotional factors, which may interfere with the echocardiography exam used to study systolic and diastolic ventricular function. The following is a detailed description of the general management of both groups. 

### 2.3. Adaptation, Incremental Exercise Test (IET), and Endurance Training Program (ETP)

**Figure 1:**
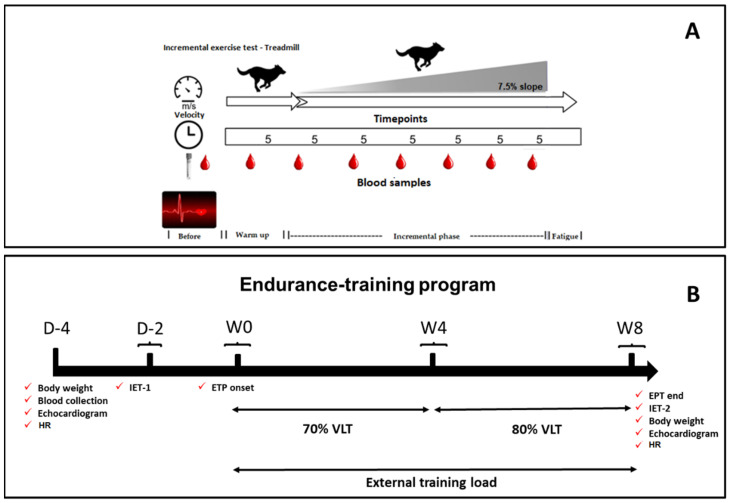
Workflow of the endurance training program (EPT). The beagle dogs were subjected to an incremental exercise test (IET-1) and their lactate threshold (LT) was determined. Treadmill slope was set to 7.5%, with 0.5 m/s speed increments, every 5 min. Blood samples were collected at the following timepoints: before the IETs and between each incremental step (**A**). Next, dogs from group T underwent an eight-week aerobic conditioning training on the treadmill (**B**) with the intensity set to 70–80% of the velocity corresponding to the lactate threshold (VLT). Then, the IET was repeated for group T (IET-2). Group C did not undergo tests or training (negative control). D = day; W = week; HR = heart rate.

An overview of the study design is shown in Figure 1. The dogs were adapted to the laboratory, where exercise testing was performed on the treadmill and the ETP. The adaptation protocol followed has been previously published by our research group [23,26]. A motorized treadmill (Galloper^®^ 5500, Sahinco) was used to perform the two incremental exercise tests (IETs) before and after (IET-1 and IET-2) the ETP, like the weekly submaximal exercise sessions during the conditioning period.

The IET (Figure 1A) was performed to determine the lactate–velocity curve (LVC) and the visual LT, establishing the lactate threshold velocity (VLT) and prescribing the individual submaximal conditioning program for group T. This protocol was adjusted from Restan et al. [25]. Once adapted to the treadmill, the dogs started the exercise test at 0% slope and 1.5 m/s initial speed during warm-up; subsequently, treadmill slope was increased to 7.5%, with 0.5 m/s speed increments, every 5 min. Between each speed increase (effort step), the mat was stopped for 2 min to collect blood samples. The velocity was increased incrementally until the dogs showed signs of fatigue, such as the inability to follow the speed of the treadmill mat. IETs were always performed in the morning when temperatures varied between 19 and 21 °C. All dogs underwent 3 h fasting, but the water was offered ad libitum. All dogs in group T were submitted to this procedure. After the conditioning period, the IET was repeated to compare the VLTs obtained in the initial and final tests and to quantify the anaerobic power and the maximum velocity (Vmax) reached in both IETs.

The ETP was performed between the IET-1 and IET-2 (Figure 1B) and lasted eight weeks. Only dogs in group T carried out the ETP, with conditioning intensity set at 70% and 80% of VLT and 7.5% slope. Thus, in the first four weeks, velocity was set at 70% of VLT and, from the 5th week on, increased to 80% of VLT. The dogs trained three times a week (alternate days) in 30 min sessions consisting of 5 min warm-up at 50% of the established VLT, 20 min exercise session at the stipulated speed (70% or 80% of the VLT), and 5 min cooling down after daily exercise at 50% of the determined speed). The dogs belonging to group C were familiarized with the researchers and the daily handling by positive reinforcements such as offering cookies and toys in the stall without forcing exercise. These activities that took place three times a week aimed at eliminating possible behavioral and emotional interferences during containment while keeping the dogs in lateral decubitus in the echocardiographic exams. Both groups had access to the identical recreation and socialization periods mentioned in item 2.1. Such kennel management is often used to maintain and promote the well-being of dogs.

### 2.4. Lactate Threshold (LT)

Between the IET’s speed increments, blood samples (2 mL) were collected from the dogs at rest in the quadrupedal position through venous catheterization of the left jugular vein, using a 16G Insyte™ catheter (Becton Dickinson and Company Belliver Industrial Estate, Plymouth, UK) that was maintained in place with a drop of Super Bonder^®^. The area was previously shaved and scrubbed with an applicator that contained 2% chlorhexidine gluconate, followed by application of topical and superficial skin anesthesia, 2.5% lidocaine and 2.5% prilocaine (EMLA^®^). Between each incremental step, the dogs rested for 2 min, and the blood sample was collected at 90 s. This procedure is based on a study previously carried out on dogs by our laboratory [26].

Venous blood samples were stored in BD Vacutainer^®^ Fluoride/EDTA tubes containing EDTA (12 mg) and sodium fluoride (6 mg) until analysis (Becton Dickinson and Company Belliver Industrial Estate, United Kingdom). The lactate concentration analysis followed the electro-enzymatic methodology (YSI 2300, Yellow Springs Instrument, Yellow Springs, OH, USA), previously validated for use in dogs [28]. LT was identified by visual inspection of LVC. Three evaluators experienced in exercise physiology examined the curves and determined the velocity at which the lactate concentration showed an abrupt and exponential increase. The inflection point represents the beginning of an imbalance between lactate production and removal/metabolism [24,25]. The VLT obtained in IET-1 was used to prescribe the individual training routine.

### 2.5. Echocardiography

The possible morphological changes and systolic and diastolic functions were evaluated by echocardiographic examination using an ultrasound system (Acuson X300 Ultrasound System, Premium Edition, Siemens, Munich, Germany) in the 5.0–7.5 MHz range to allow for both resolution and sufficient penetration. After shaving the right and left thoracic regions, the dogs were restrained in the lateral decubitus position to obtain the standard images following recommendations previously established by Boon [29] and the American Society of Echocardiography [30] for conventional and tissue echocardiography.

### 2.6. Morphological and Volumetric Evaluation

The evaluations included two-dimensional, M-mode, pulsed, continuous Doppler color, and tissue flow. The M-mode image was obtained in the chordal plane in the cross-section of the right parasternal window by positioning the cursor perpendicularly to the interventricular septum and equidistant from the papillary muscles. From this image, echocardiographic variables such as left ventricular internal diameter at the end of diastole (LVDd), left ventricular internal dimension at the end of systole (LVDs), left ventricular free wall thickness (LVW), and interventricular septum (IVS) were obtained during diastole and systole. Lastly, the fractional shortening (FS%) was determined by the Teichholz method.

The diameters of the left atrium (LA) and aorta (Ao) were measured from the 2D image at the level of the aortic valve, in the transversal image of the right parasternal window, to determine the left atrium to aorta ratio (LA/Ao). The left ventricular internal diameter at the end of diastole to aorta ratio (LVDd/Ao) and the left ventricular internal diameter at the end of systole to aorta ratio (LVDs/Ao) were determined. The 2D measurements also included left ventricular end-systolic (LVVs) and end-diastolic (LVVd) volumes, as well as ejection fraction (EF) and systolic volume (SV), which were calculated by the Simpson uniplanar method.

### 2.7. Systolic and Diastolic Evaluation

The flow rate in the pulmonary artery was determined using a pulsed Doppler. The Doppler velocities of the mitral and aortic flow were acquired in the left parasternal apical windows using cross-sections of four and five chambers, respectively. The flow velocity in the aortic artery was determined using pulsed Doppler to quantify the LV ejection time (LVE) and the LV pre-ejection period (PEP). The mitral flow was assessed in the apical section of four chambers with a sample placed at the height of the tips of the mitral leaflet. The early (E) and late (A) left ventricular filling velocities, as well as the ratio of the early and late (E/A) left ventricular filling velocities, were determined in the same way. The isovolumetric relaxation time (IVRT) was measured as the time interval between the end of the aortic flow and the beginning of the mitral influx using a pulsed Doppler. Tissue Doppler images (TDI) obtained at the site of septal and lateral insertion of the mitral annulus were used to determine the systolic excursion velocities (S’), early diastolic excursion velocities (E’), and late diastolic excursion velocities (A’) [29].

### 2.8. Speckle Tracking

The indices representing the percentage of deformation and myocardial deformation speed, the strain (SR), and strain rate (SRT), respectively, were obtained using the 2D-STE methodology, previously described by Chetboul et al. [31]. For this procedure, two-dimensional images were acquired in the right parasternal cross-section, at the height of the papillary muscles, and the images were analyzed using the optical flow algorithm in the software Syngo Velocity Vector Imaging (VVI) (SIEMENS^®^). Three consecutive cardiac cycles were collected, using continuous ECG monitoring, with a sampling rate between 50 to 90 rams/s. For myocardial screening, the endocardial border was manually marked at the end of the systole and then the epicardial border was automatically delimited by the software, being manually adjusted when necessary. Point tracking was only accepted if the software inspection and visual inspection were deemed adequate. All measurements were made at week 0 (W0) and after the conditioning period, being reassessed at week 8 (W8).

### 2.9. Intra and Interobserver Variability

A repeatability study was conducted on six animals assessed the intra and interobserver variability of the speckle tracking evaluation. These dogs were randomly reassessed using images obtained previously with a minimum of fifteen days from the first evaluation. The same observer was evaluated to calculate the intraobserver variability. The same studies were examined by a researcher blinded to the results of the first investigation to measure interobserver variability. 

### 2.10. Heart Rate

The heart rate was evaluated by a 24 h Holter test using a Cardioflash^®^ (digital-Cardios Sistemas-São Paulo, Brazil) to capture the signals of cardiac electrical activity for 24 h, following the methodology previously published by our laboratory [24,26]. The electrocardiographic tracings were processed by a specific software (Cardio Manager S540, Cardios Sistemas, Brazil). The dogs remained in the stalls during the 24 h test to minimize environmental interferences to obtain the heart rate. Therefore, HR was determined during rest before and after the conditioning period.

### 2.11. Statistical Analysis

All raw data were assessed using the Shapiro–Wilk normality test. The Mann–Whitney and Student *t*-test for unpaired samples were applied to evaluate the physical and cardiac variables at the beginning of the experiment. Moreover, the Student’s *t*-test was applied to evaluate trained dogs’ aerobic and anaerobic fitness variables, such as VLT and Vmax, respectively, body weight and the 24 h resting HR. The echocardiographic variables were analyzed using two-way analysis of variance (ANOVA), with the factors, and group ((two levels: control and trained dogs) vs. conditioning period (two moments: W0, W8)). Post hoc comparisons of the data were then performed using the Tukey–Kramer multiple comparison test. Moreover, the coefficients of variation were calculated to assess intra and interobserver measurements. The principal component analysis was performed to identify the principal components and explore the echocardiographic variables associated with the differentiation between the control and trained groups. Principal components analysis (PCA) of sample replicates were performed using the prcomp function, setting the argument scale = TRUE, from the ‘stats’ package in R. This was generated using ‘factoextra’ (v.1.0.7) and pca3d (v.0.10.2) packages. The heat map method is a practical way of evaluating grouping distances and was plotted using the R ‘pheatmap’ package (version 1.0.12) implemented in the R (v.3.6.3) using values of echocardiographic variables. Other statistical analyses were performed using the SigmaPlot software (v.12.0) at 5% significance.

## 3. Results

Table 1 summarizes the physical and cardiac variables correlated with the physical characteristics and the cardiac health of the dogs in the experimental groups before the ETP. It is noteworthy that the raw data regarding both sexes were grouped and analyzed together since their physiological characteristics were not significantly different at the beginning of the ETP. The body weight of the dogs in groups C and T did not differ during the experimental period. The cardiac physiological and echocardiographic variables revealed that the dogs of both groups were healthy. None of the dogs displayed changes in rhythm (ventricular or supraventricular arrhythmias) during the study. It is essential to inform that, at the end of the ETP, a male dog of the trained group presented trivial mitral valve regurgitation, and this condition has been monitored weekly by the research team ever since. After the tenth week, mitral regurgitation was no longer evidenced by echocardiographic examination.

The body weight of the dogs did not change at the end of the study for groups T (*p* = 0.57) and C (*p* = 0.45), as well as in the intergroup comparison (*p* = 0.108). At the end of the conditioning period, HR, obtained at rest during the 24 h test, was lower in group T (*p* < 0.001) in the intragroup check compared to group C (*p* = 0.034). This study used the velocity corresponding to the inflection point of plasma lactate concentration (VLT) to prescribe and evaluate the effect of training (Table 2). The intragroup comparison showed that group T aerobic and anaerobic fitness improved while VLT, Vmax, and VLT:Vmax were higher than IET-1, with *p* = 0.016, 0.017, and 0.003, respectively. The active control group consisted of an “untrained group” of dogs used in research previously published by our group that followed the same experimental design. Both VLT and Vmax did not differ between the IETs (Please see [23,24]).

**Figure 2:**
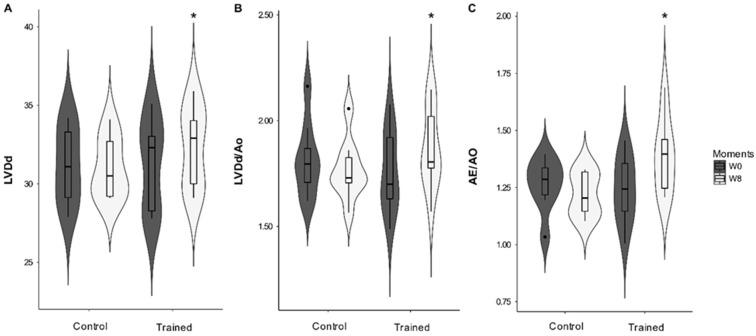
Changes in cardiac morphology of dogs submitted to eight weeks of training. (**A**) LVDd: left ventricular internal diameter at the end of diastole; (**B**) LVDd/Ao: left ventricular internal diameter at the end of diastole to aorta ratio; (**C**) LA/Ao: left atrium-to-aorta ratio. Bars are standard errors. *: Difference from week zero (W0); (*p* < 0.05).

Cardiac morphology was altered with the ETP. A significant interaction was observed between the conditioning period (moments W0 and W8) and groups (C and T) for LVDd (*p* = 0.005), DIVEd/Ao (*p* = 0.005), and LA/Ao (*p* = 0.025). DIVs and DIVs/Ao did not change significantly during the study. However, there was a statistical tendency for the conditioning period (*p* = 0.059 and *p* = 0.06, respectively) (Figure 2). The thicknesses of the interventricular septum and free wall did not change significantly.

**Figure 3:**
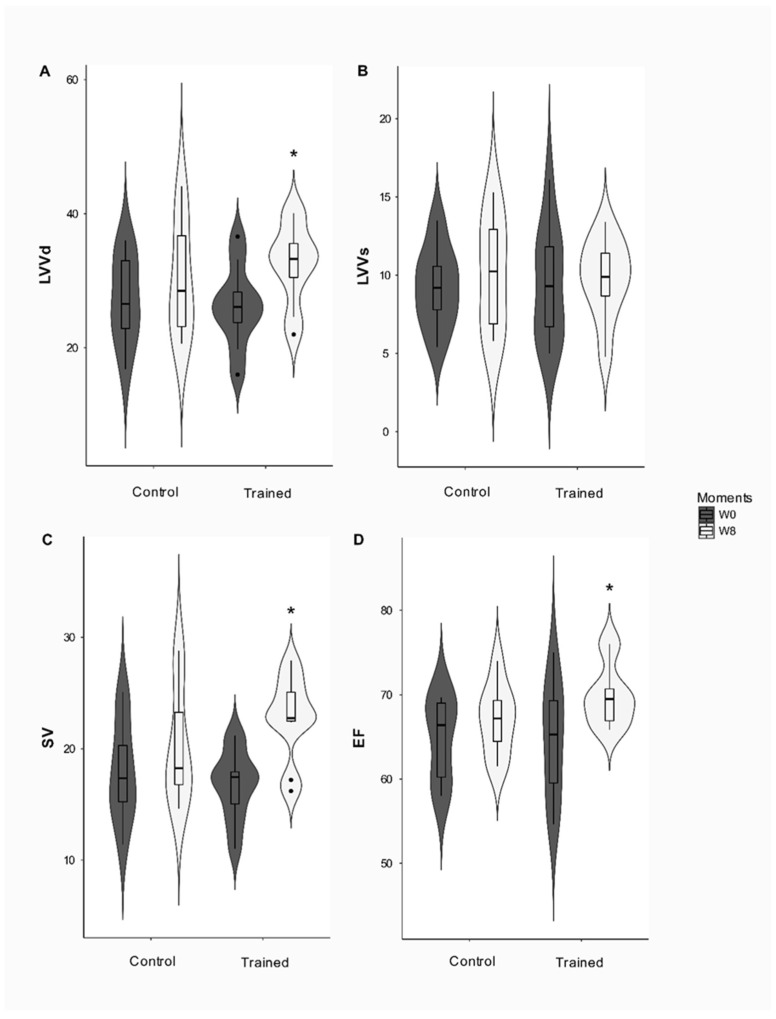
Volumetric changes in the heart in dogs submitted to 8-week endurance training program. (**A**) LVVd: left ventricular volume in diastole; (**B**) LVVs: left ventricular volume in systole; (**C**) SV: systolic volume; (**D**) EF: ejection fraction in milliliter (mL) and percentage (%). *: Difference from week zero (W0); (*p* < 0.05).

Moreover, the TEVE and PPE did not change after the conditioning period. The FS% had a group effect (*p* = 0.01), since the T and C groups differed before the study, but this difference disappeared after the conditioning period. The conditioning period significantly affected the EF and LVVd (*p* = 0.039 and *p* = 0.003, respectively) since EF (*p* = 0.032) increased in group T after the conditioning period while LVVd increased over eight weeks (*p* = 0.004). Figure 3 reveals that VS affected the interaction (*p* = 0.039), increasing in group T at the end of the study (*p* < 0.001).

**Figure 4:**
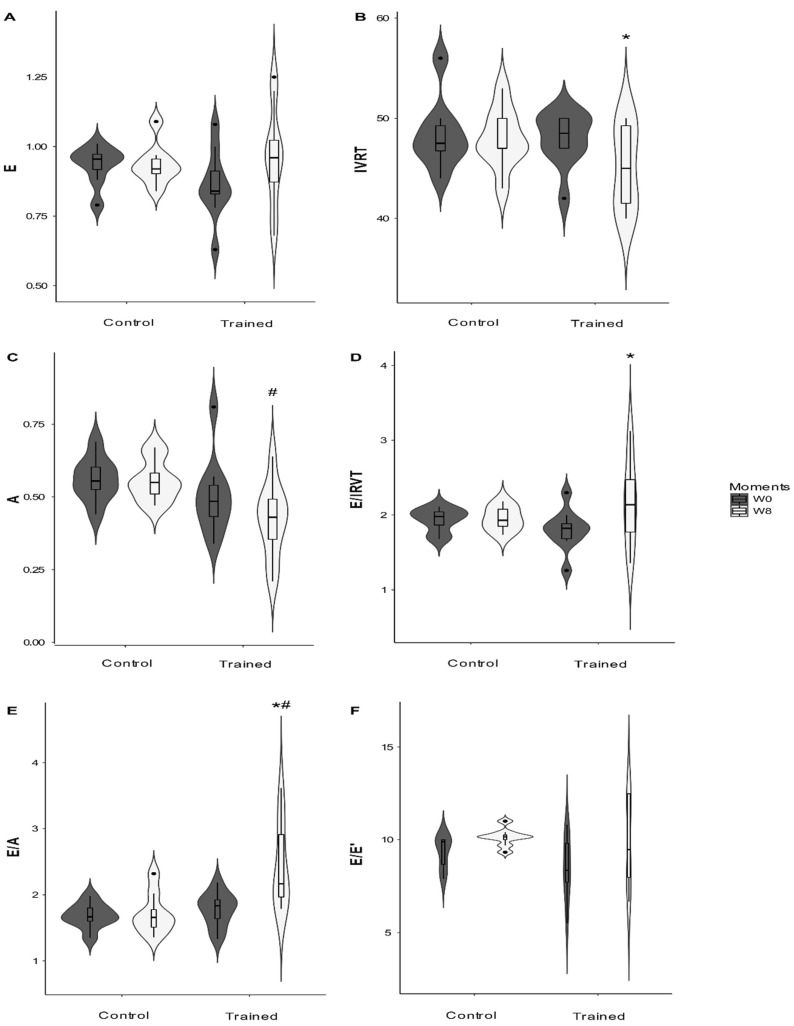
Changes in the diastolic function of dogs submitted to 8 weeks of physical training. E: the velocity of early ventricular filling velocity (**A**); IVRT: isovolumetric relaxation time (**B**); A: the velocity of late ventricular filling velocity (**C**); E/IVRT: the ratio of early ventricular filling velocity to isovolumetric relaxation time (**D**); E/A: the ratio of early-to-late left ventricular filling velocities (**E**); E/E’: ratio of the mitral flow E wave and mitral annulus E’ wave velocities (**F**). *: Difference compared to week zero (W0); #: Difference between groups (*p* < 0.05).

The tissue Doppler did not change in the study (Table 3). Diastolic function increased with conditioning. Wave E did not change but wave A had a group effect (*p* = 0.03), decreasing in group T after the conditioning period (*p* = 0.04). A significant interaction (*p* = 0.026) between the conditioning period and group was observed for the E/A ratio, which was higher in the trained group compared to group C, in W8 (*p* < 0.001), and at this moment, the intragroup comparison also indicated that this variable improved in the dogs of group T (*p* = 0.002). IVRT and E/IVRT had an effect in the period (*p* = 0.049 and *p* = 0.035, respectively). IVRT decreased in the trained group after the conditioning period (*p* = 0.012). On the other hand, E/IVRT increased in group T (Figure 4) after eight conditioning weeks (*p* = 0.005). E/E’ was also affected by the conditioning period (*p* = 0.02) and increased in group T in W8 (*p* = 0.006).

**Figure 5:**
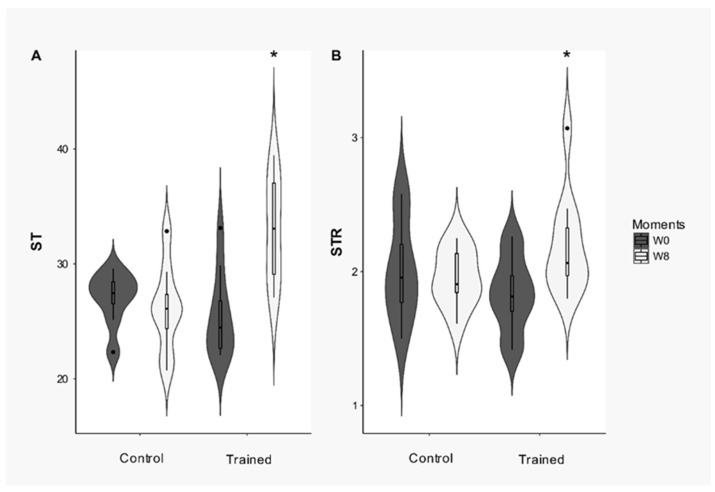
Changes in myocardial deformation (**A**) Strain and (**B**) strain rate of the left ventricle in dogs after 8 weeks of physical training. *: Difference from week zero (W0); (*p* < 0.05).

Speckle tracking was used for precisely evaluating myocardial function from the two-dimensional echocardiogram curves by the objective quantification of myocardial deformation and left ventricular systolic and diastolic dynamics. The SR and radial SRT indexes changed significantly with the ETP. The SR had a significant interaction between the conditioning period and the group (*p* = 0.001). Figure 5 shows that SR increased significantly in the trained dogs compared to the control group after the ETP (*p* < 0.001). A significant interaction between the conditioning period and group (*p* = 0.03) was observed for SRT, which increased significantly at W8 in the trained group (*p* = 0.009). Finally, the inter and intraobserver repeatability analyses showed acceptable coefficients. The SR and SRT indices demonstrated reliable intraobserver analyses, with coefficients of variation (CV) determined as 7.5% and 5.45%, respectively. The interobserver analysis had a coefficient of variation of 6% and 4.42% for SR and SRT, respectively.

**Figure 6:**
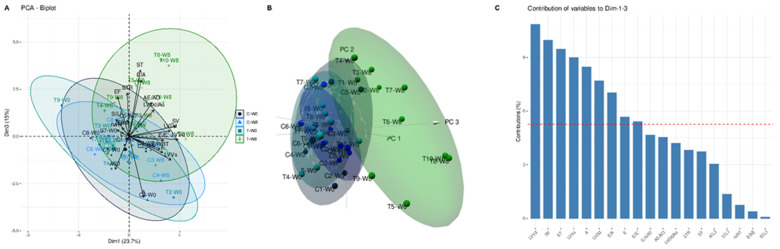
Principal component analysis (PCA) of the dogs undergoing an eight-week endurance training program. In plot (**A**), each dot represents one dog in the respective training week (W0 and W8), and echocardiographic variables are analyzed. Plot (**B**) shows scatterplots corresponding to groups (C-W0, control in week 0, purple; C-W8, control in week 8, blue; T-W0, trained in week 0, light green; T-W8, trained in week 8, dark green) and similar condition profiles cluster together. A clear separation between control and trained dogs is observed. In (**C**), the barplot reveals the echocardiographic variables’ contribution on dimensions 1 and 3. The reference red dashed line shown on the barplot corresponds to the expected value if the contribution were uniform. The variables with a contribution above the reference line could be considered the most important on the selected dimensions.

PCA characterizes and compiles a dataset to mitigate the dimensionality of the nineteen measured echocardiographic variables into three components (58.35% of the total variance). A biplot was performed to observe the variability and similarities of echocardiographic variables among the studied dogs, revealing distinct profiles for C and T groups, and their association with each variable (Figure 6A,B). In addition, from this PCA we obtained the contribution of each variable on components 1 and 3, which were selected to clearly distinguish the trained dogs (Figure 6C). The variables with a contribution above the reference value, i.e., the expected value if the contribution were uniform, could be considered as important for the related dimensions.

**Figure 7:**
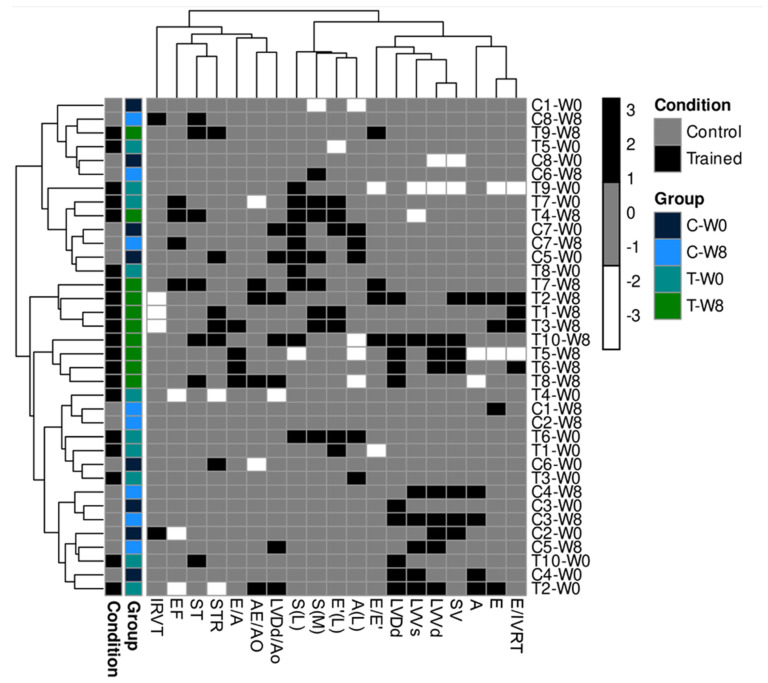
Heatmap of echocardiographic variable scores of dogs submitted to eight weeks of the endurance training program. The color gradient represents score magnitude; the blacker, the more massive the echocardiographic response.

The heatmap visualization technique is one of the most practical ways of evaluating cluster distances. The map shows the grouping of scores obtained after evaluating the echocardiographic variables of the groups before and after ETP. The black and white gradients show the contrasting scores, that is, the observed response intensities, where the white color indicates a minimal or absent response, and the black color suggests a higher response. In this sense, Figure 7 reveals that dogs trained for eight weeks were clustered in the center of the heatmap (green rectangles) with the highest intensity of black areas.

## 4. Discussion

Our study was the first to evaluate the effect of chronic submaximal exercise prescribed from the velocity corresponding to the LT on the diastolic and systolic function in beagle dogs. The main finding is that this type of aerobic conditioning improved cardiac function, with physiological adjustments characterized by ventricular dilation, increased early diastolic relaxation, and improved LV radial systolic mechanics. To date, no study has reported such changes in cardiac function at rest due to an endurance training program in dogs guided by the external load corresponding to the lactate threshold.

To study the correlation between the multiple echocardiographic variables, some complex system approaches propose detecting the coordination variables, also known as the order or collective variables, using the principal component analysis (PCA), a multivariate statistical technique for identifying the coordination variables [15]. The PCA discriminated against echocardiographic variables used for evaluating morphological changes (LVDd), probably induced by the increased volume load (LVVd, SV, and LVVs), as well as diastolic (E/A, A, E and E/E’) and systolic (ST) adjustments. Thus, the PCA and the heatmap quantified the effect induced by the ETP while capturing the resulting qualitative changes. Our method to evaluate the ETP effects provided qualitative information that complemented the traditionally used submaximal performance indexes (HRrest and VLT) and maximal index Vmax. 

The VLT, Vmax, and VLT:Vmax of trained dogs increased at the end of the eight-week training period. Likewise, previous studies with horses [32] and dogs [23,24] reported similar findings. Moreover, the VLT increase suggests less need to use the anaerobic glycolytic pathway for producing ATP, indicating an improvement of aerobic fitness. This probably resulted from the increase in the number of mitochondria and oxidative enzymes in muscle fibers, which maximized the metabolic pathway of oxidative phosphorylation [32]. Besides, the Vmax increase in dogs of the trained group observed in IET-2 represented a positive anaerobic metabolic adjustment in response to the conditioning program [23]. This latter study reported that VLT and Vmax remained unchanged in dogs not submitted to the same conditioning protocol used here compared with trained dogs. VLT-based submaximal training prescription protocol improved the oxidative functional capacity in dogs. Another aspect being addressed is the decreasing HR detected by the 24 h electrocardiography at rest. This response is expected after aerobic conditioning programs, with bradycardia used as a marker of cardiovascular improvement [24]. These results confirm that the ETP improved the aerobic and cardiovascular capacity of the trained dogs.

The increase of the LVDd, LVDd/Ao, and LA/Ao variables are consistent with other results obtained in human endurance athletes [11,20] and sled dogs after training [33]. The LA and left ventricular sizes of the trained dogs increased significantly, consistent with increased preload, where LV end-diastolic volume was the major determinant of LA volume increase in athletes [7,34]. During ventricular diastole, the LA is exposed to LV systolic pressure. Consequently, when the LV pressure increases, the LA pressure rises to maintain adequate LV filling. This increasing tension in the atrial wall can lead to dilation of the chamber and stretching of the myocardium of the LA [7,35]. In our study, both LV and LA diameters increased in the trained dogs, with a proportional increase in LV systolic volume caused by volume overload without eccentric LV hypertrophy. This finding is compatible with short-term conditioning periods and changes observed due to physical conditioning [9]. Therefore, the increased LA and LV dimensions of dogs submitted to aerobic conditioning can be considered a physiological consequence and included in the dog’s athlete’s heart context.

Adaptations induced by exercise in LV function have been relatively well studied. Studies have examined the LV systolic function of human athletes at rest [11,20,36]. In the present study, FS% did not change with regular exercise, a result compatible with a previous study with sled dogs, indicating that this cardiac variable remains unchanged after training [33]. Additionally, FS% is not commonly used to determine improvement in systolic function in humans, as it measures only radial contractility. However, in most cases, the longitudinal increments of the systolic function appear before the radial function. Thus, FS% must be interpreted from the hemodynamic point of view. It can be affected not only by the degree of myocardial contractility but also by the conditions of preload and afterload, and HR [37].

On the other hand, LVDd, SV, and EF increased in the trained dogs. The systolic function assessed by EF is generally normal among athletes of the human species [36]. This finding contradicts other studies [11,20,31,36] that reported that the practice of long-term exercise training did not cause these variables to change. However, there are reports of increased systolic function in Olympic athletes who have undergone intense and uninterrupted endurance training [38].

Still on this topic, the increase in LVDd and SV are classic findings in trained individuals [11,20,36]. SV can increase significantly with training during exercise and at rest. The increase in the cardiac chamber and the ability to generate a higher systolic volume are the direct results of the athlete’s physical training and the increase in the LV end-diastolic volume [8]. This LV end-diastolic volume is determined by diastolic filling, a complex process that is affected by a variety of variables, including heart rate, intrinsic myocardial relaxation, ventricular compliance, ventricular filling pressures, atrial contraction, among others [36].

The E/A ratio of the transmitral flow increased in the trained group, probably induced by the A wave decrease. Additionally, other parameters such as E/IVRT and E/E’ also increased in the trained dogs, while the IVRT values decreased after the conditioning period. These findings are compatible with increased LV and LA pressure, an effect induced by an increase in the final diastolic volume [39,40,41]. The decrease in IVRT to less than <45 ms, as well as the increase in the E/IVRT (values > 2.3), E/E (values > 12), and E/A (>2) ratios are strongly correlated with the increased pressure of LV filling in dogs [40,42,43]. It is highlighted that shortened IVRT, together with increased E/A, E/IVRT, and E/E (Figure 2) is, by definition, an integral part of restrictive diastolic dysfunction, a flow pattern considered specific to advanced diastolic dysfunction and high LV pressure [40,43,44]. Although, after the conditioning period, the trained group had increased E/A and reduced IVRT compared to the beginning of training (W0), while the E/IVRT and E/E’ parameters tended to the upper limit of normality, this finding cannot be interpreted as a diastolic dysfunction of the LV but as a normal finding in dogs submitted to physical conditioning.

The TDI parameters (S’, E’, and A’), measured in the septal and lateral mitral annulus, remained similar in both experimental groups. Although conventional Doppler echocardiographic variables are useful for distinguishing between physiological or pathological LV diastolic changes, the TDI results were ineffective for detecting cardiac characteristics/parameters in human athletes [11].

LV diastolic function has also been extensively assessed in trained individuals using pulsed and tissue Doppler. It is well recognized that regular exercise promotes an increase in LV early diastolic filling assessed by the E wave velocity, the velocity of the mitral annular tissue, and IVRT [10,34,45,46]. Human studies have shown that hemodynamic changes occurring during acute exercise are the primary stimulus for cardiac remodeling. Aerobic training can increase LV early diastolic filling [47] and faster heart filling during intense exercise [48]. This rapid filling is due to the mechanical forces resulting from the heart remodeling that leads to a noticeable increase in the intraventricular pressure gradient, whereby blood is rapidly sucked from the LA to the LV apex [8,49]. Therefore, this diastolic suction and rapid LV filling are mainly due to the LV’s ability to quickly relax at high heart rates during physical exercise, being an essential mechanism for preserving systolic volume during exercise [36].

Considering that the FS% of the LV remained unchanged in the dogs of the T group, more direct LV contractility indexes obtained through speckle tracking increased significantly. Specifically, the SR and SRT increased in the trained dogs while speckle tracking data revealed increased radial systolic function, despite an FS% without LV changes.

Recent advances in quantifying LV strain can help characterize myocardial contractility in a wide range of experimental and clinical settings [31,50,51,52]. The increase in SR and radial SRT resulting from fitness protocols, even when EF remains unchanged, has been elucidated in humans [11]. This methodology has been demonstrated to be more sensitive for determining systolic function changes than traditional echocardiographic indexes and is not influenced by the HR of trained individuals [11]. Similarly, previous studies on dogs have shown that speckle tracking was little or not influenced by HR [52]. Certainly, the data from the present study revealed that ETP was able to improve systolic function, as determined by speckle tracking more sensitively, even without changes observed in the conventional echocardiogram.

### 4.1. Study Limitations

Some limitations of our study must be recognized. First, the failure to perform the initial and final stress test in the active control group to demonstrate the influence of training on VLT and Vmax. However, a previous study showed that our conditioning protocol was responsible for the improved aerobic fitness of dogs when comparing active and untrained dogs [23]. Second, the lack of measurements of systolic pressure in dogs before and after the conditioning period. This variable could have complemented the results obtained here. Additionally, the lack of measurements of parameters evaluating the longitudinal systolic function since such data could have reinforced the present study’s findings. It has been shown that changes in systolic function may occur first in the longitudinal deformation parameters of the myocardium and then in the radial function of the heart [53]. Nevertheless, the findings obtained here showed an improvement in systolic function. Further studies are needed to confirm whether the use of variables to determine longitudinal systolic function effectively determines changes in the systolic function of dogs undergoing regular exercise.

### 4.2. Clinical Implications

Systolic and diastolic function is adversely altered in many pathological conditions, especially heart disease [43,54]. In cardiac patients, physical conditioning is used as nonpharmacological treatment, since they have been demonstrated to increase the quality of life and survival while decreasing cardiac remodeling, maintaining the systolic function, and improving diastolic function [55]. However, to date, the prescription of regular exercise programs in Veterinary Cardiology is still limited, especially as a treatment for diseases such as mitral valve degeneration [25]. Besides, there are clinical implications for human patients after mitral valve repair. Following mitral repair, exercise training was safe and associated with an improvement in exercise capacity, not deteriorating the outcome of recent surgery, and effective in increasing peak oxygen consumption and the anaerobic threshold [56,57]. Physical conditioning improves cardiac function (diastolic and systolic function), has positive vascular effects (angiogenesis, increase in nitric oxide production), and neurohormonal and autonomic effects such as the increase in the parasympathetic system and antiarrhythmic effects, and the decrease in angiotensin II concentrations, which are beneficial for the quality of life and survival of human patients [58]. Thus, the results of this study with healthy animals are essential for future studies, since the literature expresses the need for several controlled clinical studies using healthy patients to evaluate the results, efficacy, and safety of several training modalities [59].

It is still necessary to discuss the male dog that presented reversal of mitral valve regurgitation diagnosed at the end of the experimental period. Mitral, aortic, or pulmonary regurgitation is a relatively common clinical finding in retired human athletes, horses, and greyhounds [60,61]. This regurgitation is considered physiological, generally induced by morphofunctional adjustments caused by regular exercise, and does not represent a pathological finding in trained individuals [62]. It is important to emphasize that the alteration diagnosed here was no longer detected by conventional echocardiography after a ten-week detraining period. A study in rats showed that cardiac adjustments resulting from regular exercise could be reversed after a similar period of detraining [63]. Further studies are needed to quantify the incidence of mitral regurgitation in dogs undergoing training and to characterize the time of reversibility of these physiological adjustments.

## 5. Conclusions

Eight weeks of continuous exercise prescribed with submaximal external load promoted concomitant LV dilation without hypertrophy of the ventricular walls, emphasizing LV systolic and diastolic function. This conditioning protocol can be applied to improve systolic and diastolic functions in dogs. These findings together imply the need for future studies to confirm that the protocol used here has similar effects in dogs affected by chronic diseases that alter the cardiac function and could pave the way for new treatments for dogs with heart failure. 

## Figures and Tables

**Table 1:** Physical characteristics and echocardiography variables of dogs at the beginning of the trial.

Variable	C	T	*P*
Age (months)	12 (12–20)	14 (12–24)	0.63 ^‡^
Body weight, kg	12.4 ± 1.1	11.2 ± 1.3	0.06 *
LA:Ao	1.27 ± 0.1	1.23 ± 0.1	0.53 *
LVDd/Ao	1.83 ± 0.1	1.76 ± 0.1	0.4 *
LVDs/Ao	1.12 ± 0.1	1.15 ± 0.2	0.4 *
FS (%)	37.54 ± 6.2	34.20 ± 4.9	0.20 *
E:A	1.68 ± 0.2	1.73 ± 0.2	0.65 *
HR (bpm)	90 ± 8	97 ± 12	0.136 *

*: Student’s *t*-test; ^‡^: Mann–Whitney test. LA/Ao: left atrium to aorta ratio; LVDd/Ao: left ventricular internal dimension at end of diastole to aorta ratio; LVDs/Ao: left ventricular internal dimensions at end of systole to aorta ratio; FS: fractional shortening; E: A: ratio of early-to-late left ventricular filling velocity; HR: heart rate; C: control group; T: trained group.

**Table 2:** Pre- vs. post-training values for body weight, HR_resting_, VLT, V_max_, and VLT:V_max_.

Variable	C		T	
	W0	W8	W0	W8
Body weight (kg)	12.4 ± 1.1	12.3 ± 1.1	11.2 ± 1.3	11.28 ± 1.4
HR_resting_ (bpm)	90 ± 8	86 ± 9	97 ± 12	73 ± 14 ^#^*
VLT (m/s)	N/A	N/A	3.75 ± 0.7	4.70 ± 0.5 *
Vmax (m/s)	N/A	N/A	4.90 ± 0.7	5.65 ± 0.7 *
VLT:V_max_ (%)	N/A	N/A	75.9 ± 5.6	83.4 ± 4.0 *

C: control group; T: trained group; HR: heart rate obtained during 24 h at rest; LT: lactate threshold; Vmax: maximum speed; W0: week 0; W8: week 8; N/A, does not apply. ^#^: Indicates reduction to the C in week 8. *: Indicates intragroup increase in relation to the week 0. Please see our recently published study for changes in VLT and Vmax in the control group (untrained) after the ETP period [24].

**Table 3:** Doppler velocities of the septal and lateral mitral annulus.

Parameter	T Group		C Group	
	W0	W8	W0	W8
Septal annulus				
E’ peak (m/s)	0.10 ± 0.01	0.10 ± 0.03	0.09 ± 0.003	0.09 ± 0.003
A’ peak (m/s)	0.06 ± 0.01	0.063 ± 0.02	0.06 ± 0.01	0.07 ± 0.01
S’ peak (m/s)	0.10 ± 0.01	0.10 ± 0.02	0.096 ± 0.01	0.09 ± 0.01
Lateral annulus				
E’ peak (m/s)	0.14 ± 0.03	0.14 ± 0.02	0.13 ± 0.02	0.13 ± 0.01
A’ peak (m/s)	0.09 ± 0.01	0.08 ± 0.02	0.09 ± 0.02	0.09 ± 0.01
S’ peak (m/s)	0.13 ± 0.03	0.12 ± 0.03	0.11 ± 0.02	0.10 ± 0.03

E’: early wave to septal/lateral annular tissue velocity; A: late wave to septal/lateral annular tissue velocity; S’: systolic peak to septal/lateral annular tissue velocity; C: control group; T: trained group.

## Data Availability

The raw/processed data required to reproduce these findings are available from the authors upon request.
